# ICU Patients’ Antibiotic Exposure and Triazole-Resistance in Invasive Candidiasis: Parallel Analysis of Aggregated and Individual Data

**DOI:** 10.3389/fphar.2021.586893

**Published:** 2021-03-22

**Authors:** Yan Wang, Ying Zhang, Treasure M. McGuire, Samantha A. Hollingworth, Mieke L. Van Driel, Lu Cao, Xue Wang, Yalin Dong

**Affiliations:** ^1^ Department of Pharmacy, the Second Affiliated Hospital of Xi’an Jiaotong University, Xi’an, China; ^2^ Department of Pharmacy, the First Affiliated Hospital of Xi’an Jiaotong University, Xi’an, China; ^3^ School of Pharmacy, the University of Queensland, Woolloongabba, QLD, Australia; ^4^ Faculty of Health Sciences and Medicine, Bond University, Robina, QLD, Australia; ^5^ Mater Pharmacy, Mater Health, Brisbane, QLD, Australia; ^6^ Primary Care Clinical Unit, Faculty of Medicine, University of Queensland, Brisbane, QLD, Australia; ^7^ Department of Pharmacy, Beiguan Community Health Service Center, Xi’an, China; ^8^ Central Intensive Care Unit, the First Affiliated Hospital of Xi’an Jiaotong University, Xi’an, China

**Keywords:** invasive candidiasis, critical illness, drug resistance, fungal, time series analysis, regression analysis

## Abstract

**Background:** The relationship between antibiotic use and the incidence of triazole-resistant phenotypes of invasive candidiasis (IC) in critically ill patients is unclear. Different methodologies on determining this relationship may yield different results.

**Methods:** A retrospective multicenter observational analysis was conducted to investigate exposure to antibiotics and the incidence of non-duplicate clinical isolates of *Candida* spp. resistant to fluconazole, voriconazole, or both during November 2013 to April 2018, using two different methodologies: group-level (time-series analysis) and individual-patient-level (regression analysis and propensity-score adjusting).

**Results:** Of 393 identified *Candida* spp. from 388 critically ill patients, there were three phenotypes of IC identified: fluconazole-resistance (FR, 63, 16.0%); voriconazole-resistance (VR, 46, 11.7%); and cross-resistance between fluconazole and voriconazole (CR, 32, 8.1%). Exposure to several antibacterial agents with activity against the anaerobic gastrointestinal flora, especially third-generation cefalosporins (mainly cefoperazone/sulbactam and ceftriaxone), but not triazoles, have an immediate effect (time lag = 0) on subsequent ICU-acquired triazole-resistant IC in the group-level (*p* < 0.05). When the same patient database was analyzed at the individual-patient-level, we found that exposure to many antifungal agents was significantly associated with triazole-resistance (fluconazole [adjusted odds ratio (aOR) = 2.73] or caspofungin [aOR = 11.32] on FR, voriconazole [aOR = 2.87] on CR). Compared to the mono-triazole-resistant phenotype, CR IC has worse clinical outcomes (14-days mortality) and a higher level of resistance.

**Conclusion:** Group-level and individual-patient-level analyses of antibiotic-use-versus-resistance relations yielded distinct but valuable results. Antibacterials with antianaerobic activity and antifungals might have “indirect” and “direct” effect on triazole-resistant IC, respectively.

## Introduction

Invasive candidiasis includes both bloodstream and deep-seated invasive infections caused by *Candida* species (Martin-Loeches et al., 2019). *Candida* spp. account for up to 17% of all intensive-care unit (ICU)-acquired infections globally (Vincent et al., 2009). Half of candidemia episodes occur in the ICU setting reflecting the complexity of illness usually associated with this infection (Pappas et al., 2018). The incidence and mortality rates for IC in ICU patients have progressively increased over time (Lortholary et al., 2014); a mortality rate approaching 40% has been reported for ICU-acquired IC in most series (Calandra et al., 2016).

Echinocandins and triazoles (mainly fluconazole and voriconazole) are backbones for the treatment of IC and are recommended by several guidelines (Wang et al., 2019). Although echinocandins are increasingly regarded as first-line treatment superior to fluconazole, the echinocandin-resistant rate remains relatively low (Perlin, 2015). Triazoles are still the most wildly used antifungal agents worldwide (Guo et al., 2013; Vallabhaneni et al., 2018). Given that antifungal resistance is an emerging threat to the initiation of appropriate antifungal treatment, early recognition of risk factors for patients who may experience triazole-resistant IC can improve patient survival (Perlin et al., 2017).

Previous studies demonstrated that exposure to antibiotics, (i.e. antifungal or antibacterial agents) is a prominent factor for increasing the risk of emergence and resistance of *Candida* species (Ben-Ami et al., 2012; Martin-Loeches et al., 2014; Jensen et al., 2015). However, most of these studies were based on data from individual-patients and it is known that antimicrobial treatment may exert effects on individual-patients that are substantially different in magnitude to the effects at the population-level, (i.e. group-level) (Lipsitch and Samore, 2002). For many types of pathogens, the relationship between antibiotic usage and resistance is mediated by selection at the population-level due to patient-to-patient transmission; it also applies to *Candida* spp. in ICU (Hammarskjold et al., 2013). Therefore, adopting the perspective of both the group and individual-patient level to identify the relationship between antibiotic usage and triazole-resistant IC will inform therapeutic decision making.

The objectives of this study were to: 1) model the time-varying effect of antibiotic use, (i.e. antifungals and antibacterials; aggregated data) on the incidence of three triazole-resistant phenotypes (fluconazole-resistance [FR], voriconazole-resistance [VR], and cross-resistance between voriconazole and fluconazole [CR]) in ICU patients using autoregressive integrated moving average (ARIMA) models and a transfer function, (i.e. group-level); 2) perform individual-patient-level analyses to identify potential antibiotic-risk factors associated with the three resistant phenotypes by using the same dataset, comparing the similarities and differences of the results from two different methodologies.

## Materials and Methods

### Study Design and Patients

This retrospective study was performed at two urban, tertiary hospitals (2,700-bed and 2,200-bed, respectively), affiliated with the same university. These are the two largest tertiary hospitals in the area of central and south of Xi’an, and adopt similar medical models and practice guidelines. The ICUs of these two hospitals have 100 and 50 beds, respectively. The data collection procedures were approved by each hospital’s institutional review board. All data in this study were collected retrospectively, all patient data were anonymous and all laboratory information were part of routine testing, so informed consent was waived (No. 2015-XJTUFAH-002 and No. XJTUSAH-2020071).

Adult patients (age ≥18 years) were enrolled if diagnosed with proven IC in ICU and ICU stay ≥3 days. Proven IC was defined as a clinical illness consistent with an IC diagnosis combined with a *Candida* spp. isolate being detected by positive specimen culture taken from a sterile site (tissue or blood) or through histopathological evidence.

The four exclusion criteria were: 1) patients with neutropenia (absolute neutrophil count <500 cells/μl), hematologic malignancy, or after hematopoietic stem cell transplant; 2) microbiological isolates collected within 48 h of ICU admission; 3) being infected with rare *Candida* species whose clinical breakpoints or epidemiological cutoff-values for fluconazole and voriconazole have not been determined; and 4) incomplete data on susceptibility testing.

The details of antibiotic use, *Candida* spp. identification and susceptibility testing, and individual-patient data collection are provided in the (Supplementary Appendix SA1).

### Statistical Analysis

#### Group-Level Analysis

We determined if there were significant changes in monthly antibiotic use, the incidence of the most commonly isolated *Candida* spp., and the triazole-resistant *Candida* spp., (i.e. FR, VR and CR) using linear regression over the study period. Application of simple regression analyses would be inappropriate as the temporally sequenced observations on antibiotic use, the incidence of *Candida* spp., and the resistance isolates are not independent (Lopez-Lozano et al., 2000). Therefore, we assessed the relationship between monthly antibiotic use and monthly incidence of triazole-resistant *Candida* spp.; as well as monthly incidence of *Candida* spp. using a dynamic regression model. In the current study, this method comprises modeling the monthly incidence of *Candida* spp. or triazole-resistant *Candida* spp (explained variable) using ARIMA models with the Box–Jenkins method, integrating the stochastic dependence of consecutive data over time (Helfenstein, 1996). Then, the transfer function (TF) modeling method was employed to quantify the dynamic relationship by adding antibiotic use as an explanatory variable, and the possible time delays (lags) of up to six months was considered (Lopez-Lozano et al., 2000; Vernaz et al., 2011). This methodology has been proposed by Lopez-Lozano *et al.* as a suitable method to examine the relationship between antibiotic exposure and the emergence of drug-resistance (Lopez-Lozano et al., 2000) (See details of the statistical analysis of ARIMA and TF model in the Supplementary Appendix SA2).

### Individual-Patient-Level Analysis

A multivariate multinomial regression analysis was performed to identify risk factors for IC due to non-*albicans Candida* species, using *C. albicans* as reference.

A multivariate stepwise logistic regression model with backward selection was used to determine the relevant factors of FR, VR and CR. A *p* < 0.05 was considered statistically significant in the final multivariate model. To limit confounding by nonantibiotic-risk factors, we calculated the conditional probability of recent exposure to specific antibiotics based on nonantibiotic-risk factors using propensity-score adjusting analysis. We estimated the cumulative incidence of 14 days all-cause mortality by using the Kaplan-Meier product-limit method. The difference in cumulative incidence was compared by the log-rank test for trend and Cox regression analysis (see details of the statistical analysis of the regression model in the Supplementary Appendix SA3). All statistical analyses were performed using SAS 9.4 (SAS Inc.).

## Results

We identified 393 episodes of IC in 388 patients during the 54 months study period. Of these episodes, the median age was 63 years (IQR 46, 74 years) and, 221 (56%) patients were male. There were a median of 119 (IQR 109, 130) ICU discharges per month and the median duration of ICU stay was 11.38 days (IQR 9.74, 13.42).

### Antibiotic Use and Triazole-Resistance of *Candida* spp

Detailed antibiotic use and *Candida* spp. results are presented in the Supplementary Material (Supplementary Appendix SA4). Trends in the use of each class of antibiotics are summarized in the Supplementary Table S1 (Supplementary Appendix SA5). The distribution and the incidence of triazole-resistance of *Candida* spp. are shown in Table 1.

**TABLE 1:** Episodes of invasive candidiasis in number and incidence (per 100 patient-days) of non-duplicate *Candida* spp. distribution and resistance to fluconazole and voriconazole (November 2013 to April 2018).

Organism		2013 (from November)	2014	2015	2016	2017	2018 (until April)	Total	*P* value^a^
*C. albicans*	No. (%) of isolate	5 (45.5)	42 (55.3)	65 (67.7)	81 (71.7)	52 (63.4)	4 (26.7)	249 (63.4)	
	Incidence	0.23	0.32	0.46	0.57	0.32	0.08		0.612
*C. glabrata*	No. (%) of isolate	3 (27.3)	10 (13.2)	16 (16.7)	10 (8.8)	7 (8.5)	3 (20.0)	49 (12.5)	
	Incidence	0.14	0.08	0.11	0.07	0.04	0.06		0.157
*C. tropicalis*	No. (%) of isolate	2 (18.2)	13 (17.1)	5 (5.2)	11 (9.7)	13 (15.9)	3 (20.0)	47 (12.0)	
	Incidence	0.09	0.10	0.04	0.08	0.08	0.06		0.930
*C. parapsilosis*	No. (%) of isolate	1 (9.1)	0 (0)	3 (3.1)	2 (1.8)	5 (6.1)	2 (13.3)	13 (3.3)	
	Incidence	0.05	0	0.02	0.01	0.03	0.04		0.133
*C. krusei*	No. (%) of isolate	0 (0)	4 (5.3)	2 (2.1)	3 (2.7)	2 (2.4)	2 (13.3)	13 (3.3)	
	Incidence	0	0.03	0.01	0.02	0.01	0.04		0.688
Other *Candida* spp.	No. (%) of isolate	0 (0)	7 (9.2)	5 (5.2)	6 (5.3)	3 (3.7)	1 (6.7)	22 (5.6)	
	Incidence	0	0.05	0.04	0.04	0.02	0.02		0.430
All *Candida* spp.	No. (%) of isolate	11 (100)	76 (100)	96 (100)	113 (100)	82 (100)	15 (100)	393	
	Incidence	0.51	0.57	0.67	0.80	0.50	0.29		0.384
Antifungal resistance to									
Fluconazole	No. (%) of isolate	3 (27.3)	15 (19.7)	14 (14.6)	19 (16.8)	8 (9.8)	4 (26.7)	63 (15.9)	
	Incidence	0.14	0.11	0.10	0.13	0.05	0.08		0.126
Voriconazole	No. (%) of isolate	1 (9.1)	16 (21.1)	10 (10.4)	13 (11.5)	4 (4.9)	2 (13.3)	46 (11.6)	
	Incidence	0.05	0.12	0.07	0.09	0.02	0.04		0.009
Cross-resistance	No. (%) of isolate	0 (0)	10 (13.2)	5 (5.2)	12 (10.6)	4 (4.9)	1 (6.7)	32 (8.1)	
	Incidence	0	0.08	0.04	0.08	0.02	0.02		0.243

^a^ *p* values are generated by linear regression.

The number of species associated with a high fluconazole MIC (≥64 μg/ml) related to CR *Candida* infections (16/32, 50.0%) was higher than that of mono-resistance *Candida* infections (5/31, 16.1%, *p* = 0.004), and a non-significant trend for high MIC (≥2 μg/ml) of voriconazole (16/32 [50.0%] vs. 3/14 [21.4%], respectively, *p* = 0.070).

### Group-Level Analysis

The results of correlations between antibiotic use and *Candida* spp. distribution were presented in the Supplementary Material (Supplementary Appendixes SA6, SA7; Supplementary Table S2).

**FIGURE 1:**
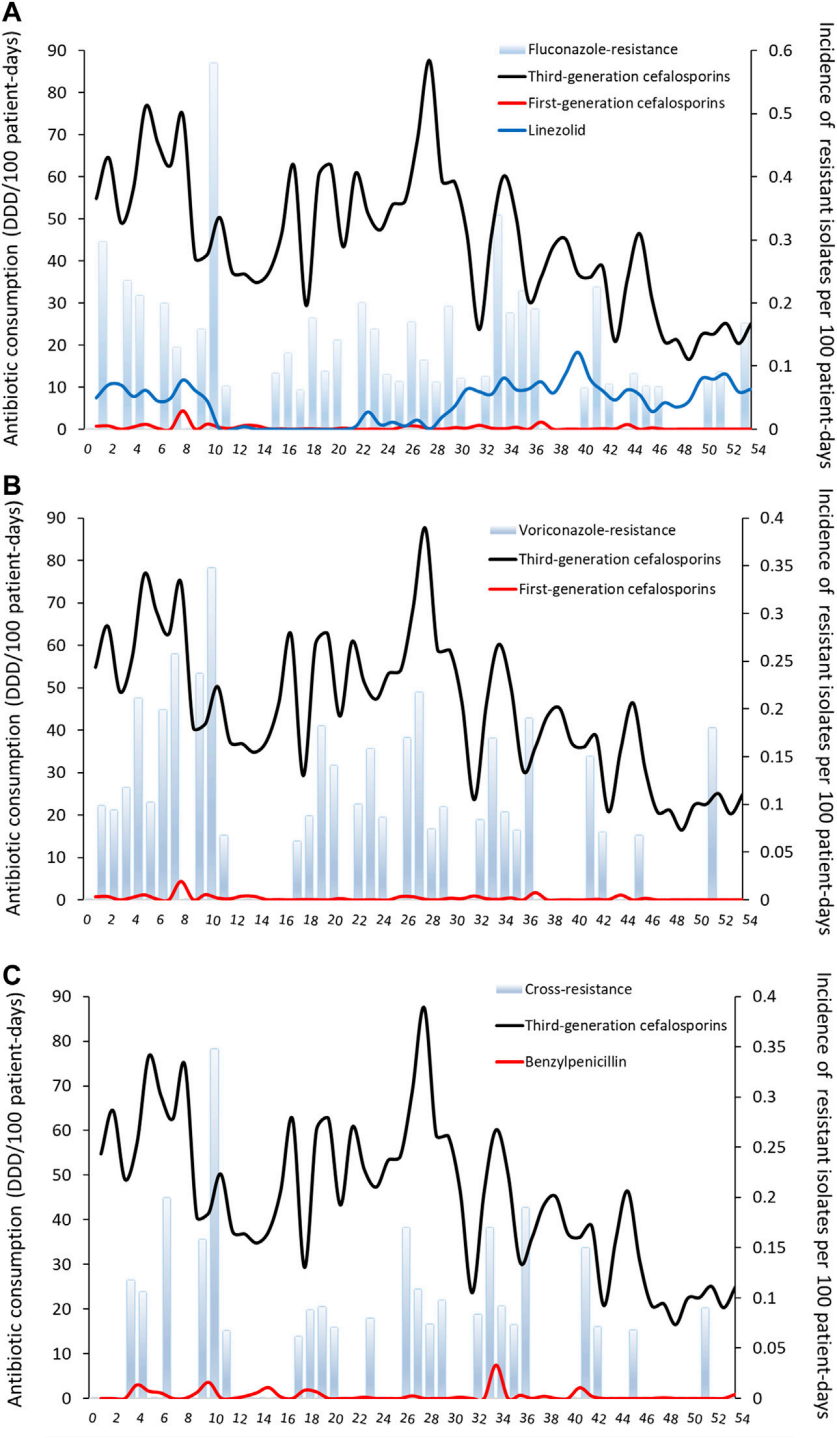
**Monthly incidence of non-duplicate triazole-resistant isolates and antibiotic consumption per 100 patient-days (A)** Fluconazole-resistant invasive candidiasis **(B)** Voriconazole-resistant invasive candidiasis **(C)** Cross-resistant invasive candidiasis. The number on the *x*-axis are months (from November 2013 to April 2018).

There were positive relationships between the incidence of FR IC and several potential explanatory variables in the dynamic regression model; for third-generation cefalosporins (mainly cefoperazone/sulbactam and ceftriaxone), first-generation cefalosporins, and linezolid. Temporal variations in third-generation cefalosporins use were followed by variations in FR IC incidence immediately, (i.e. no delay). After the third-generation cefalosporins usage increase (or decrease) by one DDD/100PD, the incidence of FR IC immediately increase (or decrease) by 0.0016/100PD. The variation of FR IC incidence was 0.083/100PD for first-generation cefalosporin (3 months delay) and 0.011/100PD for linezolid (2 months delay) (Table 2 and Figure 1A). Over the study period, half (52%) of the variations of the monthly incidence of FR IC were explained by the factors included in the model (*R* ^2^ 0.52).

**TABLE 2:** Antibiotic use for the incidence (per 100 patient-days) of non-duplicate *Candida* spp. resistant to azoles (November 2013 to April 2018) using a multivariate transfer function model.

Variable	Lag (months)	Parameter (95% confidence interval)	T Statistic	*P* value^a^
Fluconazole-resistance (*R* ^2^ = 0.52)				
Third-generation cefalosporins	0	0.0016 (0.0011,0.0021)	6.71	<0.001
First-generation cefalosporins	3	0.083 (0.051, 0.114)	5.14	<0.001
Linezolid	2	0.011 (0.003, 0.020)	2.70	0.007
Autoregressive term	5	−0.293 (−0.574, -0.013)	−2.05	0.041
Voriconazole-resistance (*R* ^2^ = 0.42)				
Third-generation cefalosporins	0	0.0016 (0.0011, 0.0020)	7.12	<0.001
First-generation cefalosporins	0	0.029 (0.006, 0.052)	2.43	0.015
Moving average term	1	−0.323 (−0.609, −0.036)	−2.21	0.027
	2	0.331 (0.046, 0.616)	2.28	0.023
Cross-resistance (*R* ^2^ = 0.30)				
Third-generation cefalosporins	0	0.0009 (0.0006, 0.0012)	6.33	<0.001
Benzylpenicillin	1	0.022 (0.009, 0.035)	3.41	<0.001
Moving average term	2	0.394 (0.135, 0.653)	2.98	0.003

^a^ *p* values are generated by multivariate transfer function model.

The incidence of VR IC was positively correlated with the previous use of third-generation cefalosporins and first-generation cefalosporins. The variation of VR was 0.0016/100PD for third-generation cefalosporins and 0.029/100PD for first-generation cefalosporins (both for no delay) (*R* ^2^ 0.42, Table 2 and Figure 1B). For CR IC, the incidence of CR IC was positively correlated with previous use of third-generation cefalosporins (0.0009/100PD, no delay) and benzylpenicillin (0.022/100PD, average delay of one month; *R* ^2^ 0.30, Table 2 and Figure 1C).

### Individual-Patient-Level Analysis

The results of multinomial regression analysis for different *Candida* species were presented in the Supplementary Material (Supplementary Appendixes SA6, SA8; Supplementary Table S3).

The demographic and clinical characteristics of the study patients (*n* = 393) and patients with different triazole-resistant phenotypes, as well as some associations for FR, VR, and CR IC identified in the bivariate analysis are presented in the Supplementary Table S4; Supplementary Appendix SA9).

The independent antibiotic-risk factors for FR IC included previous exposure to fluconazole (odds ratio [OR] 2.73, 95% confidence interval [CI] 1.38, 5.39, *p* = 0.004), caspofungin (OR 11.32, 95% CI 2.48, 51.74, *p* = 0.002), or glycopeptides (mainly vancomycin) (OR 2.32, 95% CI 1.22, 4.42, *p* = 0.010; Table 3). We found no associations with VR IC after adjusted for propensity-scores (Table 3). CR IC was positively associated with previous exposure to voriconazole only (OR 2.87, 95%CI 1.03, 8.01, *p* = 0.045) (Table 3). After adjusting for propensity-scores and SOFA score, exposure to fluconazole was associated with a non-statistically significant trend for CR IC (OR 2.26, 95%CI 0.96, 5.30, *p* = 0.062).

**TABLE 3:** Adjusted odds of antibiotics associated with fluconazole-resistant, voriconazole-resistant and cross-resistant invasive candidiasis using multivariate analyses.

Characteristic	Fluconazole-resistance		Voriconazole-resistance		Cross-resistance	
	Adjusted OR (95% CI)^c^	*p* Value^a^	Adjusted OR (95% CI)	*p* Value^a^	Adjusted OR (95% CI)^d^	*p* Value^b^
Exposure to fluconazole	2.73 (1.38, 5.39)	0.004	–	–	2.26 (0.96, 5.30)	0.062
Exposure to caspofungin	11.32 (2.48, 51.74)	0.002	–	–	–	–
Exposure to glycopeptides (mainly vancomycin)	2.32 (1.22, 4.42)	0.010	–	–	–	–
Exposure to voriconazole	–	–	–	–	2.87 (1.03, 8.01)	0.045

OR: Odds Ratio; CI: Confidence Interval; SOFA: Sequential Organ Failure Assessment.

^a^ Generated by multivariate regression analysis adjusted for antibiotic-related propensity score (*p* = 0.781).

^b^ Generated by multivariate regression analysis adjusted for antibiotic-related propensity score (*p* = 0.350) and SOFA score (*p* = 0.035).

^c^ Hosmer-Lemeshow test 64.42, *p* = 0.532.

^d^ Hosmer-Lemeshow test 81.25, *p* = 0.994.

### Clinical Outcomes

**FIGURE 2:**
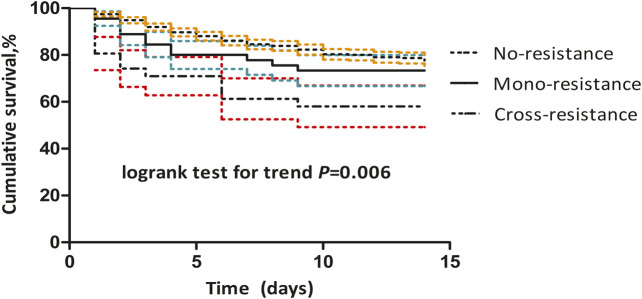
**Cumulative survival in patients with cross-resistant, mono-triazole-resistant, and non-resistant invasive candidiasis using Kaplan-Meier curves.** The dashed line indicates the 95% confidence interval: yellow for non-resistant; green for mono-triazole-resistant; red for cross-resistant invasive candidiasis.

The 14 days all-cause mortality rate was 26.0% (101/388) among the ICU patients. The rate varied: CR IC was 43.8% (14/32), mono-triazole-resistant IC was 26.7% (12/45) while those with non-resistant IC was 24.1% (75/311, *p* = 0.006; Figure 2). There was an association between 14 days all-cause mortality and resistant phenotypes (adjusted hazards ratio [aHR] 1.55, 95% CI 1.17, 2.05, *p* = 0.002) after adjusting for APACHE II scores at IC diagnosis (aHR 1.08, 95%CI 1.05, 1.11, *p* < 0.001), and invasive mechanical ventilation (aHR 1.80, 95%CI 1.10, 2.95, *p* = 0.021). There was no difference in the length of stay following infection between patients with FR IC and patients with non-FR IC, between patients with VR IC and patients with non-VR IC, nor between patients with CR IC and patients with non-CR IC (Supplementary Table S4; Supplementary Appendix SA9).

## Discussion

Exposure to antibiotic remains one of the most important risk factors for acquiring antibiotic-resistant *Candida* spp. (Ben-Ami et al., 2012). We applied two different methodologies (group-level and individual-patient-level) to analyze antibiotic-use-versus-resistance relations for *Candida* spp. isolates in critically ill patients, yielding distinct results. Exposure to several antibacterial agents, specifically third-generation cefalosporins, but not triazoles, have an immediate effect (time lag = 0) on subsequent ICU-acquired triazole-resistant IC in the group-level, indicating an "indirect" effect on resistance. When the same patient database was analyzed at the individual-patient-level, we found that exposure to many antifungal agents was significantly associated with triazole-resistance.

In group-level analyses, previous fluconazole use (for 3-months delay) was associated with the emergence of FR, VR and CR IC when using univariate TF models. However, when we introduced third-generation cefalosporins exposure (mainly cefoperazone/sulbactam and ceftriaxone) into the multivariate TF model, exposure to fluconazole was no longer a significant risk factor for developing triazole-resistant IC and the model performance became better to predict the real incidence of resistant isolates. The antibiotic-associated collateral damage which implies certain antibacterials with an antianaerobic spectrum of activity promote subsequent infection with *C. glabrata* infection (potentially resistant) (Lin et al., 2005) or FR infection (Ben-Ami et al., 2012) has been noted. *Candida* species coexists with a vast number of bacterial species in the human gastrointestinal tract, most of which are facultative or obligatory anaerobes. Most IC are caused by translocation from endogenous reservoir to normally sterile body sites. Cefoperazone/sulbactam, ceftriaxone, linezolid and benzylpenicillin, which were identified by group-level analyses for triazole-resistant IC, all have certain anti-anaerobic activity. The probable mechanism is that these antibiotics significantly alter the balance of different components of the indigenous microbial flora, which may persist for months after a relatively short course of treatment (Dethlefsen and Relman, 2011). Simply by killing off competing flora of different species, treatment with one agent may increase the burden of a pathogen resistant to another agent. For example, this has been observed with anti-anaerobic treatments that increase the load of vancomycin-resistant *Enterococci* (Donskey et al., 2000), as well as ceftriaxone, piperacillin-tazobactam, and metronidazole treatments that promote *Candida* glabrata colonization in the feces of mice (Pultz et al., 2005). Other proposed mechanisms include direct antifungal activities or modulating azole resistance by inducing the expression of efflux-pump-encoding genes of some antibacterials (de Micheli et al., 2002; Zhai et al., 2010).

Regarding the patient-level analyses, previous exposure to fluconazole or caspofungin was positively correlated with the emergence of FR IC. Given that previous exposure to echinocandins is a significant risk factor for echinocandin-resistance and multidrug resistance, our results might suggest echinocandin-resistance was strongly associated with FR, which is in agreement with previous reports (Farmakiotis et al., 2014; Pham et al., 2014). We only found that exposure to fluconazole can affect FR, but not VR and CR, while exposure to voriconazole would affect CR, but not VR. These findings were in accord with another report (Bailly et al., 2016), suggesting that we should use voriconazole carefully to avoid causing more serious drug-resistance. No triazole affected VR IC: this was unexpected and needs to be confirmed by further studies.

Particularly important findings of this study were the all-cause mortality were closely related to different resistant phenotypes (CR vs. mono-triazole-resistance vs. non-resistance) after adjusting for severity of illness, and the number of species associated with high fluconazole or voriconazole MIC was greater for CR IC than for mono-triazole-resistant IC, indicating a higher level of resistance for CR species. Altogether, CR IC may have a worrisome propensity compared to mono-triazole-resistant IC. Nevertheless, the new *Chinese medical insurance policy 2020* may still force Chinese clinicians to prioritize triazoles (mainly fluconazole and voriconazole), despite investigations showing that initial echinocandin treatment reduces mortality in critically ill patients with candidemia (Garnacho-Montero et al., 2018). This paradoxical dilemma might lead to therapeutic failure and decrease the survival of patients with IC.

Different types of epidemiological studies have been performed to quantify the association between antibiotic exposure and *Candida* resistance (Ben-Ami et al., 2012; Farmakiotis et al., 2014; Bailly et al., 2016). Our results indicated the different methodological approaches are not mutually transposable. Some experts indicated that group-level analytic methods alone cannot reliably clarify causal relationships between exposure to antibiotic and resistance due to some ecological biases, (e.g. the key information was lost in the data aggregation process-the sequence of antibiotic exposure and the emergence of a resistant organism has not been reflected). However, ecological studies (aggregated data) are potentially helpful for the researches of infectious diseases, since they can estimate the overall effect of antibiotic exposure, (i.e. not just the direct effects on the individual receiving the antibiotic but also the indirect effects mediated through effects on transmission capacity). (Harbarth et al., 2001). Therefore, considering the different ways in which antibiotics affect antimicrobial resistance, antibacterials with antianaerobic activity seem to have an “indirect” effect, while antifungal agents have a “direct” effect on triazole-resistant IC.

We acknowledge five limitations of our study. Firstly, this is a retrospective analysis and our sample size was relatively small for differentiating individual resistant *Candida* species. Inherent risk factors may differ between single species and the combined ones we used but several studies examining potential factors for resistance also treated multiple species as a single group. Secondly, the antifungal agents tested in the present study were limited to only two triazoles so multidrug resistance among different antifungal classes could not be assessed. Multidrug-resistant *Candida* species are not uncommon—a third (36%) of echinocandin-resistant isolates were also resistant to fluconazole (Pham et al., 2014). Thirdly, both the ARIMA or regression models could have missed several confounding factors, which might bias the results and lead to the observed relationships. Additionally, the stepwise regression analysis does not always choose the best possible combination of risk factors. When there is a small sample size compared to the number of variables being studied, the selection of variables using a stepwise regression will be unstable. Nevertheless, this instability is reduced when there is a sample size (or number of events) greater than 50 per candidate variable (Steyerberg et al., 2001). Moreover, by reducing the number of variables, stepwise selection will yield a simple and easily interpretable model. Fourthly, we adopted incidence as our measure for the burden of IC. It is noted that measuring the proportion of resistant isolates among all isolates is a common method, especially for situation where the overall number of infections does not change dramatically (Burton et al., 2009; Schechner et al., 2013). Nevertheless, the use of antibiotics will reduce the number of susceptible isolates, so even if the number of resistant isolates does not increase, the proportion of resistant organisms would increase. Schechner and colleagues, therefore instead recommend the “incidence” as an indicator of the burden of resistance (Schechner et al., 2013). Finally, the resistance mechanism of triazoles and defining genotype relationships between the species by molecular typing have not been clarified in our study. The clarification of these mechanisms can better help understand the level of resistance to triazoles and confirm the patient-to-patient transmission.

Despite these limitations, we believe that this study is valuable since it combines two different statistical methods (group-level and individual-patient-level) to provide further evidence on how antibiotic use influences the incidence of varying triazole-resistance in ICU patients, which allows us to interpret the results in two divergent perspectives. Moreover, we were able to investigate clinical outcomes for different resistant phenotypes to confirm the worrying status of antifungal resistance.

In conclusion, antibacterials with antianaerobic activity and antifungals might have “indirect” and “direct” effect on triazole-resistant IC, respectively, suggesting that preventing the misuse of antifungals and antibacterials with activity against the anaerobic gastrointestinal flora in ICU is necessary to avoid triazole-resistance phenomena without compromising the efficacy of antifungal therapy. Inappropriate initial treatment might lead to the more-complicated and worse cross-resistant phenotype, which should be considered when formulating healthcare policies.

## Data Availability

The raw data supporting the conclusions of this article will be made available by the authors, without undue reservation.
