# Supplementary material for: ICU Patients’ Antibiotic Exposure and Triazole-Resistance in Invasive Candidiasis: Parallel Analysis of Aggregated and Individual Data
Source: Front Pharmacol. 2021 Mar 22;12:586893. doi: 10.3389/fphar.2021.586893 (PMC8019904; doi:10.3389/fphar.2021.586893)
Supplement: Supplementary file 1 [file presentation1.pdf]

Supplementary Material

Contents

Appendix 1 The details of antibiotic use, *Candida* spp. identification and susceptibility testing, and individual-patient data collection

Appendix 2 Details of the statistical analysis of ARIMA and TF model

Appendix 3 Details of the statistical analysis of the regression model

Appendix 4 Antibiotic use, the distribution and the incidence of triazole-resistance of *Candida* spp.

Appendix 5 Quantity and trends of antibiotics use in intensive care units at First and Second Affiliated Hospital of Xi'an Jiaotong University, November 2013 to April 2018 (Table S1)

Appendix 6 Group-level and individual-patient-level analysis for *Candida* species distribution

Appendix 7 Multivariate transfer function model of antibiotic use for the incidence of non-duplicate *Candida* spp. per 100 patient-days; data from November 2013 to April 2018 (Table S2)

Appendix 8 Risk factors for invasive candidiasis in intensive care unit (N = 393) due to *C. glabrata*, *C. tropicalis*, *C. parapsilosis*, or *C. krusei* in reference to invasive candidiasis due to *C. albicans* (Table S3)

Appendix 9 Demographic and clinical features of 393 patients with invasive candidiasis infection in intensive care units (Table S4)

Appendix 1

The details of antibiotic use, *Candida* spp. identification and susceptibility testing, and individual-patient data collection

Antibiotic use

Electronic databases of hospital pharmacy records were used to extract monthly aggregated data on the use of all antibiotics (antifungal [J02 of ATC group] and antibacterial [J01 of ATC group] agents) in ICU patients from November 2013 to April 2018. We converted antibiotic doses from milligrams to defined daily doses per 100 patient-days (DDDs/100PD) (antibiotic use density), following the guidelines for ATC classification and DDD assignment (WHO Collaborating Centre for Drug Statistic Methodology; www.whocc.no).

Candida spp. identification and susceptibility testing

When multiple isolates were obtained from the same patient at the same time, only the first isolate of a given *Candida* spp. was considered in the analysis. For patients with more than one episode of candidemia, we included only the most recent infection in survival analyses. *Candida* species in specimen cultures were isolated and identified using standard microbiological procedures. We tested antifungal susceptibility using the Clinical and Laboratory Standards Institute (CLSI) microbroth dilution method to determine the minimum inhibitory concentrations (MIC) of fluconazole and voriconazole (1). In line with clinical practice, patients who were infected by at least one non-fluconazole-sensitive, non-voriconazole-sensitive or a non-wild type phenotype to fluconazole or voriconazole isolates were classified as the resistant group. The CBPs and ECVs for antifungal susceptibility were extracted from published literature to define resistance (2, 3), as summarized previously (4).

Individual-patient data collection

We used a standardized data collection template to individually review patients' medical records to collect data on demographic characteristics, concomitant diseases, time at risk (the number of days from admission to culture positive for *Candida* spp.), disease severity scores at IC diagnosis [Acute Physiology and Chronic Health Evaluation II (APACHE II) and Sequential Organ Failure Assessment (SOFA) scores], clinical outcomes (e.g. 14-day all-cause mortality and length of stay following infection) and the presence of any risk factors in the month preceding IC (e.g. surgery or central venous catheter). Additionally, we collected a detailed history of exposure to any antibiotics and the number of prior antibiotic exposures in the month preceding IC (only antibiotics that have been exposed for more than 3 days are considered).

References

1. Clinical and Laboratory Standards Institute. 2012. Reference method for broth dilution antifungal susceptibility testing of yeasts, 4th ed, M27-S4. CLSI, Wayne, PA.
2. Pfaller MA, Diekema DJ. 2012. Progress in antifungal susceptibility testing of Candida spp. by use of Clinical and Laboratory Standards Institute broth microdilution methods, 2010 to 2012. J Clin Microbiol 50:2846-56.
3. Espinel-Ingroff A, Pfaller MA, Bustamante B, Canton E, Fothergill A, Fuller J, Gonzalez GM, Lass-Florl C, Lockhart SR, Martin-Mazuelos E, Meis JF, Melhem MS, Ostrosky-Zeichner L, Pelaez T, Szeszs MW, St-Germain G, Bonfietti LX, Guarro J, Turnidge J. 2014. Multilaboratory study of epidemiological cutoff values for detection of resistance in eight Candida species to fluconazole, posaconazole, and voriconazole. Antimicrob Agents Chemother 58:2006-12.

4. Wang Y, Yang Q, Chen L, Liu L, Hao R, Zhang T, Wang X, Lei J, Xie J, Dong Y. 2017. Cross-resistance between voriconazole and fluconazole for non-albicans Candida infection: a case-case-control study. Eur J Clin Microbiol Infect Dis 36:2117-2126.

Appendix 2

Details of the statistical analysis of ARIMA and TF model

To meet the stationarity requirement (constant mean, variance and autocorrelation through time), transformed by first-order differentiation was performed if needed.

We used a three-stage model-building strategy (identification, estimation, and checking) to build ARIMA and TF models for each antibiotic-*Candida* spp. pair. Firstly, we fitted univariate ARIMA models of antibiotic use, the incidence of all or resistant *Candida* isolates series. After obtaining the univariate ARIMA models, we estimated the cross-correlations of the series to identify any significant and relevant delayed association over time (i.e. time lags). Then the antibiotic-use series (explanatory variable) at different time lags found in the last step was entered in the explained variable model using the TF model. The augmented Dickey-Fuller test for unit roots was used for transformations and differentiation diagnosis series. Secondly, we estimated parameters of the identified tentative model using an unconditioned least squares method. We used significance tests for parameter estimates with a *P* value of 0.05 to eliminate the unnecessary terms. We chose the best model with the fewest parameters and highest biological plausibility (the lowest AIC value). We estimated goodness-of-fit using the determination coefficient (R^2), which corresponds to the proportion of the variance of the observed time-series explained by the model. Thirdly, we checked residuals that effectively corresponded to white noise.

Appendix 3

Details of the statistical analysis of the regression model

Categorical variables were expressed as proportions and continuous data as medians (interquartile range [IQR]). The Mann-Whitney U test or Kruskal-Wallis test was performed for continuous variables and χ^2 or Fisher exact test for categorical variables to examine relevant risk factors, as appropriate.

In order to identify risks factors for IC due to non-*albicans Candida* species, a multivariate multinomial regression analysis was performed using *C. albicans* as reference.

Covariates with a $P<0.10$ in the bivariate analysis were considered candidates for the building of multivariable models. A multivariate stepwise logistic regression model with backward selection was used to identify factors associated with FR, VR and CR. A $P<0.05$ was considered statistically significant in the final multivariate model. If a covariate affected the β coefficient of a variable in the model by >10%, then the confounding variable was kept in the multivariate model. To limit confounding by nonantibiotic-risk factors, we calculated the conditional probability of recent exposure to specific antibiotics based on nonantibiotic-risk factors using propensity-score adjusting analysis. A propensity-score of receiving any antibiotic-risk factor was estimated using a logistic regression model that included variables with $P<0.10$ in the bivariate analysis. The propensity-score for receiving any antibiotic-risk factor was then used as a covariate in a multivariate analysis to adjust for potential confounding factors associated with antibiotic therapy. The final multivariate models were tested for overfitting using the Hosmer–Lemeshow goodness-of-fit tests. The Kaplan-Meier product limit method was used to estimate the cumulative incidence of 14-day all-cause mortality. The difference in cumulative incidence was compared by the log-rank test for trend and Cox regression analysis.

Appendix 4

Antibiotic use, the distribution and the incidence of triazole-resistance of *Candida* spp.

Fluconazole was the most frequently prescribed antifungal agent (24.75 DDDs/100PD, 55.8%), followed by voriconazole (13.03 DDDs/100PD, 29.4%), caspofungin (5.39 DDDs/100PD, 12.2%), and amphotericin B (1.17 DDDs/100PD, 2.6%). The third generation of cefalosporin (45.08 DDDs/100PD, 28.0%) and carbapenems (40.28 DDDs/100PD, 25.0%) were the most widely used antibacterial agents. During the study period, the use of fluconazole decreased (*P*=0.007) while voriconazole (*P*=0.013) and caspofungin (*P*=0.016) increased, although the overall consumption of antifungal agents remained stable. In addition, use of many antibacterial agents has significantly increased (including carbapenems, sulfamethoxazole-trimethoprim, fluoroquinolones, and linezolid) or decreased (including first-, second- and third-generation cefalosporins, macrolides, aminoglycosides, and imidazole derivatives).

Among all episodes, *C. albicans* predominated (n=249; 63%), followed by *C. glabrata* (n=49;13%), *C. tropicalis* (n=47; 12%), *C. parapsilosis* (n=13; 3%), *C. krusei* (n=13; 3%) and other *Candida* species (n=22; 6%), including *C. guilliermondii*, *C. lusitaniae*, *C. kefyr*, *C. dubliniensis* and *C. pelliculosa*. The incidence of the *Candida* species was stable over time (*P*>0.05).

We detected 63 strains of FR *Candida: C. albicans* (n=22, 35%); followed by *C. krusei* (inherent FR species) (n=13, 21%); *C. tropicalis* (n=12, 19%); *C. glabrata* (n=8, 13%); and other *Candida* species. We identified 46 VR *Candida* strains: *C. albicans* (n=18, 39%); *C. tropicalis* (n=13, 28%); *C. glabrata* (n= 5, 11%); and other *Candida* species. One in twelve (8.14%) of the population was infected by a CR species (n=32), with *C. albicans* and *C.*

tropicalis most prevalent (n= 11, 34%), followed by *C. glabrata* (n=3, 9%) and other species.

Only VR strains decreased over time (*P*=0.009).

Appendix 5

Table S1. Quantity and trends of antibiotics use in intensive care units at First and Second Affiliated Hospital of Xi'an Jiaotong University, November 2013 to April 2018

Antimicrobial class (ATC group)	Average monthly use in DDDs/100 patient-days (range)	Trend	*P* value[*]
Amphotericin B[a] (J02AA01)	1.17 (0–5.03)	No	0.116
Fluconazole (J02AC01)	24.75 (11.13–42.97)	Downward	0.007
Voriconazole (J02AC03)	13.03 (1.71–24.33)	Upward	0.013
Caspofungin (J02AX04)	5.39 (0.74–9.74)	Upward	0.016
Antifungals for systemic use, total (J02)	44.34 (27.85–65.44)	No	0.948
Tetracyclines-Tigecycline (J01AA12)	0.08 (0–2.05)	No	0.589
Penicillins with extended spectrum (J01CA)	0.02 (0–0.98)	No	0.294
β-Lactamase-sensitive penicillins-Benzylpenicillin (J01CE01)	0.60 (0–7.51)	No	0.208
Combinations of penicillins including β-lactamase inhibitors (J01CR)	12.78 (1.54–26.51)	No	0.565
First-generation cefalosporins (J01DB)	0.37 (0–4.40)	Downward	0.025
Second-generation cefalosporins (J01DC)	10.71 (4.65–24.36)	Downward	0.022
Third-generation cefalosporins (J01DD)	45.08 (16.55–87.57)	Downward	<0.001
Monobactams (J01DF)	0.16 (0–1.32)	No	0.270
Carbapenems (J01DH)	40.28 (19.07–71.43)	Upward	<0.001
Combinations of sulfonamides and trimethoprim including derivatives- Sulfamethoxazole-trimethoprim (J01EE01)	3.72 (0.20–11.18)	Upward	0.007
Macrolides (J01FA)	1.69 (0–6.81)	Downward	0.001
Lincosamides-Clindamycin (J01FF01)	0.53 (0–4.10)	No	0.642
Streptomycins (J01GA)	0.09 (0–1.25)	No	0.158
Other aminoglycosides (J01GB)	1.43 (0–6.69)	Downward	0.004
Fluoroquinolones (J01MA)	20.13 (10.26–33.34)	Upward	0.047
Glycopeptide (J01XA)	11.30 (1.15–31.64)	No	0.109
Imidazole derivatives (J01XD)	5.93 (0.08–18.44)	Downward	0.201
Other antibacterials-Linezolid (J01XX08)	6.31 (0–18.30)	Upward	0.005
Antibacterials for systemic use, total (J01)	161.20 (112.28–216.46)	No	0.707

[a] Includes liposomal amphotericin B. [*]*P* values are generated by linear regression.
DDD: Defined Daily Doses.

Appendix 6

Group-level and individual-patient-level analysis for *Candida* species distribution

Correlations between antibiotic use and Candida spp. distribution

We found many significant associations between previous use of antibiotics and the incidence of overall *Candida species* and the five most common *Candida species* (Supplementary Material Table S2, Appendix 7). Increased fluconazole use correlated significantly with the increased incidence of *C. albicans* isolates; amphotericin B for *C. tropicalis* and *C. krusei*, and voriconazole *for C. parapsilosis*.

Multinomial regression analysis for different Candida species

Using *C. albicans* as a reference, those who were more likely to be infected with *C. glabrata* were older, females, those with solid tumors, or who had previously used fluconazole and aminoglycosides. Those who were more susceptible to *C. tropicalis* infection were younger, males, those with a high APACHE II score, or who had previously used aminoglycosides. There was an increased likelihood of infection by *C. krusei* in patients with recent exposure to fluconazole. We found no associations with *C. parapsilosis* infection (Supplementary Material Table S3, Appendix 8).

Appendix 7

Table S2. Multivariate transfer function model of antibiotic use for the incidence of non-duplicate *Candida* spp. per 100 patient-days; data from November 2013 to April 2018.

Variable	Lag (months)	Parameter (SE)	T statistics	*P* value*
C. albicans (R^2=0.29)				
Fluconazole	3	0.012 (0.002)	7.38	<0.001
Aminoglycosides	5	0.061 (0.017)	3.53	<0.001
C. tropicalis (R^2=0.22)				
Amphotericin B	0	0.017 (0.007)	2.33	0.024
Combinations of penicillins including β-lactamase inhibitors	0	0.004 (0.001)	4.78	<0.001
C. krusei (R^2=0.13)				
Amphotericin B	0	0.009 (0.005)	2.02	0.048
Sulfamethoxazole	0	0.003 (0.002)	2.02	0.048
C. glabrata (R^2=0.51)				
Tigecycline	0	0.088 (0.027)	3.33	0.002
	3	0.082 (0.028)	2.88	0.006
Benzylpenicillin	2	0.016 (0.006)	2.50	0.016
Aztreonam	3	0.078 (0.029)	2.68	0.010
Constant	-	0.039 (0.010)	3.77	<0.001
C. parapsilosis (R^2=0.16)				
Voriconazole	1	0.002 (0.000)	4.48	<0.001
All *Candida* spp. (R^2=0.18)				
Autoregressive term	2	0.321 (0.143)	2.24	0.030
Aztreonam	3	0.246 (0.114)	2.16	0.036
Constant	-	0.569 (0.055)	3.06	<0.001

*P values are generated by multivariate transfer function model.

Appendix 8

Table S3. Risk factors for invasive candidiasis in intensive care unit (N = 393) due to *C. glabrata*, *C. tropicalis*, *C. parapsilosis*, or *C. krusei* in reference to invasive candidiasis due to *C. albicans*

	C. glabrata			***C. tropicalis***			***C. parapsilosis***			***C. krusei***		
	Adj. OR	95% IC	P^*	Adj. OR	95% IC	P^*	Adj. OR	95% IC	P^*	Adj. OR	95% IC	P^*
Age	**1.090**	**1.005–1.191**	**0.024**	**0.964**	**0.945–0.985**	**0.001**	0.985	0.950–1.022	0.429	0.982	0.939–1.027	0.421
Male gender	**0.438**	**0.217–0.886**	**0.022**	**2.260**	**1.072–4.761**	**0.032**	2.722	0.693–10.693	0.152	1.209	0.322–4.539	0.778
APACHE II score at diagnosis	1.074	0.990–1.166	0.085	**1.087**	**1.004–1.176**	**0.040**	1.124	0.978–1.291	0.099	1.109	0.971–1.1268	0.128
SOFA score at diagnosis	0.867	0.749–1.003	0.055	1.036	0.899–1.194	0.623	0.788	0.593–1.1046	0.099	1.079	0.827–1.409	0.574
Solid tumor	**2.449**	**1.158–5.180**	**0.019**	0.727	0.291–1.818	0.496	0.422	0.050–3.528	0.426	1.143	0.270–4.843	0.856
Septic shock	2.405	0.858–6.741	0.095	0.540	0.140–2.089	0.372	2.196	0.381–12.651	0.379	0.141	0.008–2.599	0.187
Invasive mechanical ventilation	0.785	0.378–1.629	0.516	0.529	0.254–1.101	0.089	0.407	0.121–1.375	0.148	0.558	0.123–2.526	0.449
Fluconazole	**4.006**	**1.682–9.537**	**0.002**	2.176	0.827–5.727	0.115	0.758	0.089–6.429	0.799	**8.124**	**1.952–33.812**	**0.004**
Voriconazole	0.710	0.125–4.038	0.699	2.665	0.838–8.473	0.097	2.650	0.416–16.902	0.302	2.546	0.334–19.394	0.367
Second-generation cefalosporins	1.105	0.471–2.594	0.818	0.333	0.106–1.049	0.060	0.458	0.053–3.968	0.478	3.196	0.729–14.009	0.123
Combinations of sulfonamides and trimethoprim including derivatives	1.961	0.242–15.899	0.528	0.783	0.121–5.065	0.797	2.208	0.175–27.833	0.540	-	-	-
Aminoglycosides	**2.352**	**1.060–5.219**	**0.035**	**4.832**	**2.228–10.480**	**<0.001**	0.439	0.052–3.670	0.447	0.761	0.083–7.014	0.810
Fluoroquinolones	1.822	0.862–3.848	0.116	1.308	0.604–2.836	0.496	0.866	0.213–3.525	0.841	-	-	-
Glycopeptides	0.803	0.314–2.054	0.648	1.281	0.533–3.078	0.580	1.035	0.237–4.515	0.964	4.092	0.972–17.226	0.055

OR: Odds Ratio; CI: Confidence Interval; APACHE: Acute Physiology and Chronic Health Evaluation; SOFA: Sequential Organ Failure Assessment.
Boldface indicates statistically significant associations. $^*P$ values are generated by multinomial regression analysis.

Appendix 9

Table S4. Demographic and clinical features of 393 patients with invasive candidiasis infection in intensive care units

Characteristics	Resistant type				Comparison (*P* value*)		
	Total (n=393)	FR (n=63)	VR (n=46)	CR (n=32)	FR vs. non-FR	VR vs. non-VR	CR vs. non-CR
Age (years) [median(IQR)]	63 (46–74)	62 (45–72)	67 (52–75)	63 (50–80)	0.187	0.416	0.121
No. (%) male	221 (56)	39 (62)	30 (65)	22 (69)	0.322	0.191	0.136
Time at risk (days) [median(IQR)]	11 (5–22)	10 (5–21)	11 (7–22)	17 (12–53)	**0.005**	**0.016**	**0.041**
Length of stay following infection (days) [median(IQR)]	12 (6–26)	12 (6–26)	10 (4.5–28)	11 (5–21)	0.237	0.528	0.141
Within 2 weeks before or at diagnosis *Candida* colonization, n (%)	100 (25)	21 (33)	13 (28)	11 (34)	0.117	0.641	0.226
Disease severity scores at diagnosis [median(IQR)]							
APACHE II score	16 (11–21)	16 (11–20)	17 (10–23)	19 (13–23)	0.498	0.089	0.422
SOFA score	8 (5–11)	8 (5–11)	8 (6–12)	8 (5–10)	0.179	0.070	0.080
Concomitant diseases [no. (%)]							
Lung disease	272 (69)	43 (68)	38 (83)	25 (78)	0.857	**0.036**	0.254
Diabetes mellitus	201 (51)	28 (44)	22 (48)	13 (41)	0.246	0.632	0.214
Cardiovascular disease	228 (58)	36 (57)	28 (61)	17 (53)	0.878	0.676	0.559
Chronic renal disease	119 (30)	23 (37)	15 (33)	12 (38)	0.240	0.714	0.354
Chronic hepatic disease	110 (28)	17 (27)	15 (33)	11 (34)	0.846	0.458	0.401
Solid tumor	95 (24)	19 (30)	10 (22)	7 (22)	0.226	0.682	0.751

Solid organ transplant recipient	20 (5)	2 (3)	0 (0)	0 (0)	0.659	0.189	0.344
Clinical risk factors [no. (%)]							
Diarrhea	49 (13)	9 (14)	7 (15)	3 (9)	0.634	0.548	0.784
Sepsis	71 (18)	12 (19)	12 (26)	9 (28)	0.825	0.132	0.123
Septic shock	43 (11)	7 (11)	7 (15)	6 (19)	0.962	0.323	0.140
Surgery	234 (60)	37 (59)	25 (54)	16 (50)	0.886	0.445	0.251
Dialysis	76 (19)	10 (16)	7 (15)	6 (19)	0.447	0.451	0.930
Gastrointestinal perforation	283 (72)	46 (73)	31 (67)	23 (72)	0.846	0.458	0.986
Urinary catheter	273 (70)	40 (64)	32 (70)	21 (66)	0.261	0.988	0.623
Central venous catheter	238 (61)	38 (60)	29 (63)	23 (72)	0.966	0.714	0.172
Indwelling arterial catheter	40 (10)	7 (11)	6 (13)	3 (9)	0.789	0.671	1.000
Invasive mechanical ventilation	263 (67)	45 (71)	35 (76)	25 (78)	0.407	0.160	0.160
Total parental nutrition	379 (96)	62 (98)	46 (100)	32 (100)	0.581	0.335	0.524
Non–antimicrobial drug exposure [no. (%)]							
Proton pump inhibitor	298 (76)	45 (71)	32 (70)	21 (66)	0.374	0.291	0.160
Any corticosteroid	156 (40)	34 (54)	22 (48)	16 (50)	**0.012**	0.230	0.214
Immunosuppressant	7 (2)	1 (2)	0 (0)	0 (0)	1.000	1.000	1.000
Chemotherapy	6 (2)	1 (2)	0 (0)	0 (0)	1.000	1.000	1.000
Antibiotic exposure [no. (%)]							
Fluconazole	67 (17)	21 (33)	14 (30)	11 (34)	**<0.001**	**0.010**	**0.007**
Voriconazole	29 (7)	8 (13)	6 (13)	6 (19)	0.134	0.206	**0.027**
Caspofungin	8 (2)	5 (8)	1 (2)	1 (3)	**0.002**	1.000	0.496
Tetracyclines	11 (3)	4 (6)	3 (7)	2 (6)	0.148	0.249	0.223
Benzylpenicillin	2 (1)	1 (2)	1 (2)	1 (3)	0.295	0.221	0.156
Combinations of penicillins including	233 (59)	39 (62)	28 (61)	19 (59)	0.644	0.816	0.992

β-lactamase inhibitors							
Second-generation cefalosporins	80 (20)	11 (18)	5 (11)	3 (9)	0.533	0.089	0.107
Third-generation cefalosporins	76 (19)	15 (24)	12 (26)	7 (22)	0.327	0.217	0.705
Carbapenems	206 (52)	34 (54)	27 (59)	18 (57)	0.788	0.364	0.651
Sulfamethoxazole	14 (4)	6 (10)	2 (4)	2 (6)	**0.016**	1.000	0.720
Macrolides	2 (1)	0 (0)	0 (0)	0 (0)	1.000	1.000	1.000
Aminoglycosides	80 (20)	15 (24)	10 (22)	5 (16)	0.458	0.804	0.488
Fluoroquinolones	108 (28)	23 (37)	18 (39)	12 (38)	0.080	0.060	0.185
Glycopeptides	74 (19)	22 (35)	11 (24)	10 (31)	**<0.001**	0.348	0.061
Imidazole derivatives	76 (19)	12 (19)	8 (17)	6 (19)	0.949	0.722	0.930
Linezolid	45 (12)	5 (8)	5 (11)	3 (9)	0.339	0.895	0.924
Number of antibiotics exposed to [no. (%)]					**0.006**	0.293	0.315
0	9 (2)	1 (2)	1 (2)	1 (3)			
1	74 (20)	8 (13)	5 (11)	4 (13)			
2	110 (28)	13 (21)	11 (24)	7 (22)			
3	80 (20)	9 (14)	9 (20)	5 (16)			
≥4	120 (31)	32 (51)	20 (44)	15 (47)			
Candida species					–	–	–
Candida albicans	249 (63)	22 (35)	18 (39)	11 (34)			
Candida glabrata	49 (13)	8 (13)	5 (11)	3 (9)			
Candida tropicalis	47 (12)	12 (19)	13 (28)	11 (34)			
Candida parapsilosis	13 (3)	2 (3)	1 (2)	1 (3)			
Candida krusei	13 (3)	13 (21)	2 (4)	2 (6)			
Candida guilliermondii	6 (2)	2 (3)	2 (4)	1 (3)			
Candida lusitaniae	5 (1)	2 (3)	2 (4)	1 (3)			

Candida kefyr	5 (1)	1 (2)	1 (2)	1 (3)
Candida dubliniensis	3 (1)	0 (0)	1 (2)	0 (0)
Candida pelliculosa	3 (1)	1 (2)	1 (2)	1 (3)

APACHE: Acute Physiology and Chronic Health Evaluation; CR: cross-resistance; FR: fluconazole-resistance; SOFA: Sequential Organ Failure Assessment; VR: voriconazole-resistance.

Boldface indicates statistically significant associations.

[*] *P* values are generated by bivariate analysis.
